# Supplementary material for: SeroBA(v2.0) and SeroBAnk: a robust genome-based serotyping scheme and comprehensive atlas of capsular diversity in Streptococcus pneumoniae
Source: Microb Genom. 2025 Oct 17;11(10):001483. doi: 10.1099/mgen.0.001483 (PMC12534171; doi:10.1099/mgen.0.001483)
Supplement: Uncited Supplementary Material 1. [file mgen-11-01483-s001.pdf]

Introduction

This document contains summary tables describing the genetic basis for determining the new serotypes and subtypes added to the SeroBA CTVdb. It is intended to be used in addition to the original PneumoCAT reference determination document which was last updated in 2019. SeroBA still uses all the genetic determinants from this document for determining the serotypes described in the PneumoCAT reference determination document.

This information is also available in the yaml files in the SeroBA GitHub repository (https://github.com/sanger-bentley-group/seroba)

This document contains summary tables describing the genetic and structural differences for the new serotypes and subtypes added to the CTVdb.

Feedback is welcome, you can get in touch via the GitHub repository and you can customise the CTVdb to suit your own needs.

Table of Contents

Introduction *1*

New Serotype Summary Table........ 3

New subtype summary table 5

Serogroup 11........ 7
Serogroup 15........ 7
Serogroup 20........ 7
Serogroup 24........ 7
Genogroup 33A/33E/33F/37 8
Genogroup 33G/33H 8
Serogroup 36........ 8
Serotype 6A 8
Serotype 6B 8
Serotype 19A 9
Serotype 19F 9
Serotype 33F 10
Serotype Reference sequence information 10
Subtype reference sequence information 10
Non encapsulated reference strains 11

References 11

New Serotype Summary Table

Serogroup	Serotype	Distinguishing genetic features	Functional effect
11	11A/11B/11C/11D/11E/11F/11F_like	Genes *wcwC* and *wcjE* are present in 11A, 11D, 11E and 11F_like, whereas *wcwR* is present in 11B and 11C.	*wcwC*, *wcjE* and *wcwR* are acetyl transferases – differences in acetylation
	11A/11B/11C/11D/11E/11F/11F_like	Frameshift mutation delA 130 in *gct* in 11B and 11F causes inactivation of *gct* gene	Presence of Gro-1P correlates with intact *gct* gene (Mavrodi et al. 2007)
	11A/11D/11E/11F/11F_like	SNPs in *wcrL* (Table 1)	Donor sugar for *wcrL* is GlcpNAc in types 11F, 11B and 11C, Glcp in 11A (Mavrodi et al. 2007)
	11F_like	Divergent *wcrL* and *wcwC* genes	*wcwC* changes not understood yet, lower glycosyltransferase activity in *wcrL* compared to 11F (Manna et al. 2018)
15	15A/15B/15C/15D/15F	15F has 4 additional genes: *glf, rmlB*, *rmlD* and *wcjE*	*glf*, *rmlB* and *rmlD* are involved in sugar biosynthesis. *wcjE* is an acetyltransferase
	15A/15B/15C/15D	15A/15D *wzd* has ~70% identity in the last 300bps compared to *wzd* 15B/15C	*wzd* is involved in translocation of the CPS to the cell surface, thus determining the length of the capsule polysaccharide chain
	15B/15C	Difference in TA tandem repeat near position 413 in *wciZ*, leading to frameshift in 15C (Bentley et al. 2006)	Differences in acetylation<br>15B, 15C and 15B/C results can be assigned
	15A/15D	Difference in *wzy* allele – 15A *wzy-15A*. 15D – *wzy-15D* (a *wzy-15AF* hybrid)	Different polymerization linkages (Mavrodi et al. 2007)

20	20A/20B	Insertion in polyA tract in *whaF* near position 881 leads to truncated gene in 20A	Loss of glycosyltransferase function in 20A (Calix et al. 2012)
	20C	Disruptive mutation in *wciG* leads to truncated gene	Loss of O-acetyl transferase function in 20C (unpublished)
24	24A / 24B/24C/24F	24A lacks *rbsF* gene present in 24B/24C/24F	Loss of ribofuranose biosynthesis in 24A (Ganaie et al. 2021)
33 and 37	33A/33E/33F/37	37 carries *tts* (a transferase gene)	*tts* is responsible for polysaccharide capsule biosynthesis (Waite et al. 2003)
	33A/33F	Frameshift mutation insT 433 in *wcjE* gene	Loss of acetyltransferase function leads to differences in acetylation (Mavrodi et al. 2007)
	33B/33D	*wciN*α in 33B, *wciN*β in 33D	*wciN*α codes for a glycosyltransferase, *wciN*β codes for a galactosyltransferase
	33A/33E/33F	Nonsense mutation in *wciE*, around position 273 C->T truncates *wciE* in 33E	Inactivated glycosyltransferase gene in 33E (Ganaie et al. 2023)
33G/33H	33G/33H	33H has a disruptive mutation in *wciG* causing inactivation	Loss of O-acetyl transferase function in 33H (unpublished)
36	36A/36B	*wcjA*α in 36A, *wcjA*β in 36B. SNPs in *wcjA* (Table 6)	Mutations lead to the incorporation of Glcp in 36A vs Galp in 36B (Ganaie et al. 2023)

New subtype summary table

Serotype	Subtypes	Distinguishing genetic features	Functional effect
6A	6A/6A-I/6A-II/6A-III/6A-IV/6A-V/6A-VI	Except for 6A-IV, different allelic combinations define each subtype within the 6A serotype	Nonsynonymous mutations in all subtypes leading to varying levels of structural divergence from the 6A reference sequence
	6A-I	3 divergent alleles: *wzg*α, *rmlA-3* and *rmlB-4*	
	6A-II	3 divergent alleles: *wzg*β, *wzy*α and *rmlA-2*	
	6A-III	2 divergent alleles: *wzg*χ and *rmlA-4*	
	6A-IV	SNP in *wze* pos 488	
	6A-V	2 divergent alleles: *wzg*α and *rmlA-5*	
	6A-VI	4 divergent alleles: *wzg*β, *wzy*α, *rmlA-2* and *rmlC-2*	
6B	6B/6B-I/6B-II	Different allelic combinations define each subtype within the 6B serotype	Nonsynonymous mutations in all subtypes leading to varying levels of structural divergence from the 6B reference sequence
	6B-I	3 divergent alleles: *wzg*χ, *rmlA-4* and *rmlB-3*	
	6B-II	3 divergent alleles: *wzg*δ, *rmlA-5* and *rmlB-3*	
19A	19A/19A-I/19A-II/19A-III/19AF	Different allelic and mutational combinations define each subtype within the 19A serotype	Nonsynonymous mutations in all subtypes leading to varying levels of structural divergence from the 19A reference sequence
	19A-I	2 divergent rhamnose alleles: *rmlB-2* and *rmlD-2*	

	19A-II	2 divergent rhamnose alleles: *rmlB-2* and *rmlD-2*. SNP in *wzg* differentiates from 19A-I	
	19A-III	SNP in *wzd* pos 154 determines 19A-III	
	19AF	19AF has 19F *wzy* allele	19AF phenotype as 19F despite having overall 19A-like capsular operon sequence
19F	19F/19F-I/19F-II/19F-III/19F-IV	Different allelic and mutational combinations define each 19F subtype	Nonsynonymous mutations in all subtypes leading to varying levels of structural divergence from the 19F reference sequence
	19F-I	SNPs in *wze*	
	19F-II	SNPs in *wze* and divergent wchA-4 allele	
	19F-III	SNPs in *wchO*, *wzx* and *wzy*	
	19F-IV	3 divergent alleles: *wchA-4*, *rmlB-6* and *wzg-2*	

Sequences for the above table were obtained from Elberse et al. 2011. See Table 14.

Serogroup 11

11F_like was previously typed as 11A by SeroBA

Table 1

Gene	variant	11A	11B	11C	11D	11E	11F	11F_like
wcwC	detected	Y	N	N	Y	Y	Y	Y
wcjE	detected	Y	N	N	Y	Y	Y	Y
wcwR	detected	N	Y	Y	N	N	N	N
gct	pseudo (130delA)	N	Y	N	N	N	Y	N
wcrL	pos 49	TCA	-	-	TCA	TCA	TCA	CCA
wcrL	pos 334	AAT	-	-	ACT	AAT	GCT	AAT
wcrL	Pos 493	GAC	-	-	GAC	GAC	GAC	AAT

Serogroup 15

15D was previously typed as 15F or 15A by SeroBA

Table 2

Gene	variant	15A	15B	15C	15D	15F
wzd	allele	wzdα	wzdβ	wzdβ	wzdα	-
glf	detected	N	N	N	N	Y
rmlB	detected	N	N	N	N	Y
rmlD	detected	N	N	N	N	Y
wcjE	detected	N	N	N	N	Y
wciZ	pseudo	-	N, [412,417]	Y, [412,417]	-	-
wzy	allele	wzyα	-	-	wzyβ	-

Serogroup 20

Serogroup 20 isolates were typed at the serogroup level only in the old version of SeroBA.

Table 3

Gene	variant	20A	20B	20C
whaF	pseudo	Y	N, [881,882]	N, [881,882]
whaF	pseudo	N	N	N
wciG	pseudo	N	N	Disruptive mutation

Serogroup 24

Serogroup 24 isolates were previously typed as “serogroup 24”. 24A can now be distinguished due to the absence of the rbsF gene. The genetic determination of 24B/C/F was deemed too difficult due to a lack of knowledge about the specific genetic determinants (mutations) for these serotypes.

Table 4

Gene	variant	24A	24B/24C/24F
rbsF	detected	Y	N

Genogroup 33A/33E/33F/37

33E was previously typed as 33F in SeroBA.

Table 5

Gene	variant	33A	33E	33F	37
tts	detected	N	N	N	Y
wcjE	pseudo	N, [[433, 434], T]]	-	Y, [[433, 434], TA]]	-
wciE	pseudo	N, [[273, 274, C]]	Y, [[273, 274, T]]	N, [[273, 274, C]]	-

Genogroup 33G/33H

Table 6

Gene	variant	33G	33H
wciG	pseudo	N	Disruptive mutation

Serogroup 36

In the previous version of SeroBA, serogroup 36 samples were serotyped at the serogroup level only.

Table 7

Gene	variant	36A	36B
wcjA	allele	wcjAα	wcjAβ
wcjA	pos 430	[AGT, S]	[GGT, G]
wcjA	pos 805	[TTT, F]	[ATG, M]
wcjA	pos 808	[GGC, G]	[GCT, A]
wcjA	pos 817	[GCT, A]	[GTT, V]

Serotype 6A

Samples which are typed as 6A now undergo subtyping to highlight the genetic diversity within the 6A serotype. Samples are only subtyped if all allelic and mutational criteria are satisfied.

Table 8

Gene	variant	6A	6A-I	6A-II	6A-III	6A-IV	6A-V	6A-VI
wzg	allele	wzgγ	wzgα	wzgβ	wzgχ	wzgγ	wzgα	wzgβ
rmlA	allele	rmlA-1	rmlA-3	rmlA-2	rmlA-4	rmlA-1	rmlA-2	rmlA-4
rmlB	allele	rmlB-1	rmlB-4	rmlB-1	rmlB-1	rmlB-1	rmlB-1	rmlB-1
rmlC	allele	rmlC-1	rmlC-1	rmlC-1	rmlC-1	rmlC-1	rmlC-1	rmlC-2
wzy	allele	wzyγ	wzyγ	wzyα	wzyγ	wzyγ	wzyγ	wzyα
wze	pos 488	[GCT, A]	[GCT, A]	[GCT, A]	[GCT, A]	[GTT, V]	[GCT, A]	[GCT, A]

Serotype 6B

Samples which are typed as 6B now undergo subtyping to highlight the genetic diversity within the 6B serotype. Samples are only subtyped if all allelic and mutational criteria are satisfied.

Table 9

Gene	variant	6B	6B-I	6B-II
wzg	allele	wzgε	wzgχ	wzgδ
rmlA	allele	rmlA-3	rmlA-4	rmlA-5
rmlB	allele	rmlB-4	rmlB-3	rmlB-3

Serotype 19A

Samples which are typed as 19A now undergo subtyping to highlight the genetic diversity within the 19A serotype. Samples are only subtyped if all allelic and mutational criteria are satisfied. In some cases, it is not possible to determine the SNPs which differentiate 19A-I from 19A-II, when this occurs the type called is 19A-I/19A-II.

Table 10

Gene	variant	19A	19A-I	19A-II	19A-III	19AF
rmlB	allele	rmlB-1	rmlB-2	rmlB-2	rmlB-1	rmlB-1
rmlD	allele	rmlD-1	rmlD-2	rmlD-2	rmlD-1	rmlD-1
wzy	allele	wzy-1	wzy-1	wzy-1	wzy-1	wzy-2
wzg	pos 445	[GCT, A]	[TCT, S]	[GCT, A]	[GCT, A]	[GCT, A]
wzg	pos 484	[AAT, N]	[AAT, N]	[GAC, D]	[AAT, N]	[AAT, N]
wzd	pos 154	[ACC, T]	[ACC, T]	[ACC, T]	[ATC, I]	[ACC, T]

Serotype 19F

Samples which are typed as 19F now undergo subtyping to highlight genetic diversity. Samples are only subtyped If all allelic and mutational criteria are satisfied.

Table 11

Gene	variant	19F	19F-I	19F-II	19F-III	19F-IV
rmlB	allele	rmlB-5	rmlB-5	rmlB-5	rmlB-5	rmlB-6
wchA	allele	wchA-3	wchA-3	wchA-4	wchA-3	wchA-4
wzg	allele	wzg-1	wzg-1	wzg-1	wzg-1	wzg-2
wchO	pos 67	[AGT, S]	[ATT, I]	[AGT, S]	[ATT, I]	[AGT, S]
wze	pos 135	[GAG, E]	[AGG, R]	[GGG, G]	[GAG, E]	[GGG, G]
wze	pos 207	[ATC, I]	[ATC, I]	[ATC, I]	[ATC, I]	[CTC, L]
wze	pos 477	[ATT, I]	[GTT, V]	[ATT, I]	[ATT, I]	[ATT, I]
wzx	pos 1111	[ATA, I]	[ATG, M]	[ATA, I]	[ATG, M]	[ATA, I]
wzx	pos 1133	[ATA, I]	[CTA, L]	[CTA, L]	[CTA, L]	[CTA, L]
wzy	pos 213	[ATT, I]	[CTT, L]	[ATT, I]	[CTT, L]	[ATT, I]

Serotype 33F

Table 12

Gene	variant	33F	33F-1a	33F-1b
wcjE	detected	Y	N	N
wchO	pseudo	NA	N	Y

Serotype Reference sequence information

Table 13

Serotype	Accession number	Strain	Reference
**11F_like**	MF140334	PMP1342	Manna et al. 2018
**15D**	SAMN14150919	20184245	Pimenta et al. 2021
**20A**	JQ653094	6320	Calix et al. 2012
**20B**	JQ653093	5931-06	Calix et al. 2012
**20C**	ERS653863	GPS_NP_6772	Unpublished
**24C**	MW683289	MNY576	Ganaie et al. 2021
**33E**	SAMEA2203953	GPSC3	Ganaie et al. 2023
**33H**	ERS464499	GPS_ZA_887	Unpublished
**36A**	CR931708	1095/39	Bentley et al. 2006
**36B**	MK606436	GPS_NP_1196	Ganaie et al. 2023

Subtype reference sequence information

Table 14

Subtype	Accession number	Reference
**6A-I**	JF911487	Elberse et al. 2011
**6A-II**	JF911488	Elberse et al. 2011
**6A-III**	JF911497	Elberse et al. 2011
**6A-IV**	JF911492	Elberse et al. 2011
**6A-V**	JF911491	Elberse et al. 2011
**6A-VI**	JF911490	Elberse et al. 2011
**6B-I**	JF911498	Elberse et al. 2011
**6B-II**	JF911499	Elberse et al. 2011
**19A-I**	JF911512	Elberse et al. 2011
**19A-II**	JF911514	Elberse et al. 2011
**19A-III**	JF911520	Elberse et al. 2011
**19F-I**	JF911525	Elberse et al. 2011
**19F-II**	JF911524	Elberse et al. 2011
**19F-III**	JF911522	Elberse et al. 2011
**19F-IV**	JF911528	Elberse et al. 2011

**33F-1a**	GPS_US_PATH361 8	Ganaie et al. 2023
**33F-1b**	MK606435	Ganaie et al. 2023

Non encapsulated reference strains

NCC1_pspK_non_encapsulated- JF489996
NCC3_aliD_non_encapsulated - JF490008
NCC2_aliC_aliD_non_encapsulated - HE651292
NCC2_S_mitis_aliC_aliD_non_encapsulated - HE651274

References

Bentley, S.D. *et al.* (2006) 'Genetic analysis of the capsular biosynthetic locus from all 90 pneumococcal serotypes', *PLoS Genetics*, 2(3). doi:10.1371/journal.pgen.0020031.

Calix, J.J. *et al.* (2012) 'Biochemical, genetic, and serological characterization of two capsule subtypes among streptococcus pneumoniae serotype 20 strains', *Journal of Biological Chemistry*, 287(33), pp. 27885–27894. doi:10.1074/jbc.m112.380451.

Elberse, K. *et al.* (2011) 'Sequence diversity within the capsular genes of streptococcus pneumoniae serogroup 6 and 19', *PLoS ONE*, 6(9). doi:10.1371/journal.pone.0025018.

Ganaie, F.A., Saad, J.S., Lo, S.W., McGee, L., Bentley, S.D., *et al.* (2023) 'Discovery and characterization of pneumococcal serogroup 36 capsule subtypes, serotypes 36A and 36B', *Journal of Clinical Microbiology*, 61(4). doi:10.1128/jcm.00024-23.

Ganaie, F.A. *et al.* (2023) 'Novel pneumococcal capsule type 33E results from the inactivation of glycosyltransferase WCIE in vaccine type 33F', *Journal of Biological Chemistry*, 299(9), p. 105085. doi:10.1016/j.jbc.2023.105085.

Ganaie, F. *et al.* (2021) 'Structural, genetic, and serological elucidation of streptococcus pneumoniae serogroup 24 serotypes: Discovery of a new serotype, 24c, with a variable capsule structure', *Journal of Clinical Microbiology*, 59(7). doi:10.1128/jcm.00540-21.

Manna, S. *et al.* (2018) 'A novel genetic variant of streptococcus pneumoniae serotype 11a discovered in Fiji', *Clinical Microbiology and Infection*, 24(4). doi:10.1016/j.cmi.2017.06.031.

Mavroidi, A. *et al.* (2007) 'Genetic relatedness of the *streptococcus pneumoniae* capsular biosynthetic loci', *Journal of Bacteriology*, 189(21), pp. 7841–7855. doi:10.1128/jb.00836-07.

Pimenta, F. *et al.* (2021) 'New Pneumococcal Serotype 15D', *Journal of Clinical Microbiology*, 59(5). doi:10.1128/jcm.00329-21.

Waite, R.D. *et al.* (2003) ‘Spontaneous sequence duplications within capsule genes cap8e and TTS control phase variation in streptococcus pneumoniae serotypes 8 and 37’, *Microbiology*, 149(2), pp. 497–504. doi:10.1099/mic.0.26011-0.
